# Evaluation of a countrywide implementation of the world health organisation surgical safety checklist in Madagascar

**DOI:** 10.1371/journal.pone.0191849

**Published:** 2018-02-05

**Authors:** Michelle C. White, Linden S. Baxter, Kristin L. Close, Vaonandianina A. Ravelojaona, Hasiniaina N. Rakotoarison, Emily Bruno, Alison Herbert, Vanessa Andean, James Callahan, Hery H. Andriamanjato, Mark G. Shrime

**Affiliations:** 1 Department of Medical Capacity Building, Mercy Ships, Port of Toamasina, Madagascar; 2 Department of Medical Capacity Building, Mercy Ships, Africa Bureau, Cotonou, Benin; 3 University of Tennessee Health Science Center College of Medicine, Memphis, TN, United States of America; 4 Program in Global Surgery and Social Change, Department of Global Health and Social Medicine, Harvard Medical School, Boston MA, United States of America; 5 The Austin Hospital, Melbourne, Australia; 6 Directeur du Partenariat, Ministère de la Santé Publique, Antananarivo, Madagascar; 7 Department of Otolaryngology, Harvard Medical School, Boston, MA, United States of America; 8 Office of Global Surgery and Health, Massachusetts Eye and Ear Infirmary, Boston, MA, United States of America; Massachusetts General Hospital, UNITED STATES

## Abstract

**Background:**

The 2009 World Health Organisation (WHO) surgical safety checklist significantly reduces surgical mortality and morbidity (up to 47%). Yet in 2016, only 25% of East African anesthetists regularly use the checklist. Nationwide implementation of the checklist is reported in high-income countries, but in low- and middle-income countries (LMICs) reports of successful implementations are sparse, limited to single institutions and require intensive support. Since checklist use leads to the biggest improvements in outcomes in LMICs, methods of wide-scale implementation are needed. We hypothesized that, using a three-day course, successful wide-scale implementation of the checklist could be achieved, as measured by at least 50% compliance with six basic safety processes at three to four months. We also aimed to determine predictors for checklist utilization.

**Materials and methods:**

Using a blended educational implementation strategy based on prior pilot studies we designed a three-day dynamic educational course to facilitate widespread implementation of the WHO checklist. The course utilized lectures, film, small group breakouts, participant feedback and simulation to teach the knowledge, skills and behavior changes needed to implement the checklist. In collaboration with the Ministry of Health and local hospital leadership, the course was delivered to 427 multi-disciplinary staff at 21 hospitals located in 19 of 22 regions of Madagascar between September 2015 and March 2016. We evaluated implementation at three to four months using questionnaires (with a 5-point Likert scale) and focus groups. Multivariate linear regression was used to test predictors of checklist utilization.

**Results:**

At three to four months, 65% of respondents reported always using the checklist, with another 13% using it in part. Participant’s years in practice, hospital size, or surgical volume did not predict checklist use. Checklist use was associated with counting instruments (p< 0.05), but not with verifying: patient identity, difficult intubation risk, risk of blood loss, prophylactic antibiotic administration, or counting needles and sponges.

**Conclusion:**

Use of a multi-disciplinary three-day course for checklist implementation resulted in 78% of participants using the checklist, at three months; and an increase in counting surgical instruments. Successful checklist implementation was not predicted by participant length of medical service, hospital size or surgical volume. If reproducible in other countries, widespread implementation in LMICs becomes a realistic possibility.

## Introduction

In 2009, the World Health Organization (WHO) launched its 2^nd^ Global Patient Safety Challenge: Safe Surgery Saves Lives [1]. This included introduction of the WHO Surgical Safety Checklist designed to improve the quality and the safety of surgical care. Using the checklist improved compliance in six basic safety processes—confirmation of patient identity and the surgical procedure, assessment of difficult intubation risk, assessment of the risk of major blood loss, antibiotic prophylaxis given within 60 minutes of skin incision, the use of a pulse oximeter, and counting sponges and instruments—from 33% to 66% of the time. This resulted in significant reductions in mortality (1.5% to 0.8%) and morbidity (17% to 11%), with larger patient gains in lower resource settings [2].

Since 2009, numerous studies have confirmed the effectiveness of the checklist in reducing mortality and complications [3–8]. Approximately 313 million surgical operations are performed annually worldwide, with an estimated mortality rate of 5–10% and complication rate of 3–17% [9, 10]. Improving surgical care through quality and safety initiatives is therefore high on the public health agenda. Low-cost, low-tech solutions, such as the checklist, have the potential to promote widespread improvement of surgical outcomes in low- and middle-income countries (LMICs).

That the checklist works is now known. The question, then, is *how* to implement the checklist. Of 85 anesthetists interviewed from national referral hospitals in 5 different East African countries, only 25% regularly use the checklist [11]. Successful implementation requires strong leadership, flexibility, and teamwork [12]. In LMICs, implementation is further hindered by lack of knowledge or resources. For example, in sub-Saharan Africa, patient identity bands are rarely used, 70% of operating rooms do not have pulse oximeters [13]; and nurses often do not have robust systems for counting needles, instruments and sponges. Despite these challenges, there are reports of successful checklist implementation in LMICs [14–18]. However, these are limited to single hospital sites and often rely on significant commitments of time and resources from high-income country (HIC) providers, which limits wide-scale implementation.

Wide-scale national implementation of the checklist has, to date, only been reported in HICs [5, 7, 19]. Since the greatest improvements in patient outcomes occur in LMICs, finding solutions to wide-scale implementation in LMICs is essential. Furthermore, studies report a dose effect of the checklist, meaning even partial implementation can provide improvements in patient outcome, although the largest reductions in major complications occur when surgical teams complete all parts of the checklist [3, 6, 8].

Mercy Ships operates the world’s largest non-governmental hospital ship, the *Africa Mercy*, which usually stays in a given country for ten months, providing free surgeries and training. Mercy Ships has trained single hospitals in checklist implementation in Guinea and Republic of Congo and has reported sustained results at 18 months [20, 21]. Based on these experiences and those in two pilot hospitals in Madagascar, we developed a three-day checklist implementation course suitable for countrywide scale-up. The course utilizes a multi-disciplinary team approach, allowing local ownership through checklist adaptation to the local environment. It employs real-life simulation, teaches surgical counting, and provides the necessary implementation resources such as pulse oximeters. We hypothesized that, using the three-day multi-disciplinary training program, we could successfully achieve wide-scale implementation of the checklist, as measured by at least 50% compliance with the six basic safety processes at three to four months. We also aimed to determine predictors for checklist utilization.

## Materials and methods

The study was approved by the Mercy Ships Institutional Review Board (MS 2015–0004) and approved by the Madagascar Ministry of Health.

### Implementation strategy

We used a blended educational implementation strategy as defined by Powel et al [22]. The actors, actions, action targets, temporality, dose, implementation outcome and justification of our implementation strategy [23] are shown in Table 1.

**Table 1:** Specification of implementation strategy.

Domain	Strategy
Actors	Mercy Ships and Ministry of Health, Madagascar
Action	Teach surgical team how to adapt and use the WHO surgical safety checklist in their local context using a 3-day multidisciplinary educational course
Targets of the action	All individual members of the surgical team in each of the regional referral hospitals in Madagascar. Specifically targeting the individual knowledge, skills and behaviour change necessary to carry out the defined action
Temporality	6 month period of preparation (March–August 2015) 7 month period of education delivery and follow up (September 2015 –March 2016)
Dose	3 day course per hospital, with telephone follow up at 6 weeks.
Implementation outcome	Use of the checklist ‘in full’ at least 50% of the time as measured by self-reported use of 6 basic safety process measures
Justification	Prior research showing: a) Effective use of a short course designed with widespread scale-up in mind piloted at a single institution [21] b) Multidisciplinary team training is important in checklist implementation [20] Education course designed to overcome known barriers to implementation [24, 25].

### Initial needs assessment and preparation

Madagascar is divided into 22 regions with an estimated population of 24 million people. In collaboration with the Madagascar Ministry of Health, a plan was developed to implement the checklist in all the regional referral hospitals. Two regional hospitals had already received checklist training as pilot hospitals so were excluded, as was the capital region because it was part of another project, leaving 19 regional hospitals to receive checklist training. The Ministry of Health also suggested 2 district hospitals because one was large enough to function like a regional referral hospital and the other was on an island with a large tourist population. Therefore a total of 21 hospitals, in 19 regions were selected (**Table 2**).

**Table 2:** Regions, population sizes and hospitals visited.

Region	Population size* (2014) [26]	Hospital
Alaotra Mangoro	1,108,000	Ambatondrazaka
Amoron’i Mania*	772,000	Ambositra*
Analanjirofo	1,117,000	Fenerive Est
Androy	792,000	Ambovombe
Anosy	725,000	Tolagnaro
Atsimo-Andrefana	1,421,000	Toliara
Atsimo-Atsinanana	970,000	Farafangana
Betsiboka	317,000	Maevatanana
Bongolava	494,000	Tsiroanomandidy
Diana	755,000	Antsiranana, Nosy Be (2 hospitals)
Ihorombe	337,000	Ihosy
Itasy	791,000	Miarinarivo
Matsiatra Ambony	1,294,000	Fianarantsoa
Melaky	313,000	Maintirano
Menabe	639,000	Morondava
Sava	1,058,000	Sambava, Antalaha (2 hospitals)
Sofia	1,346,000	Antsohihy
Vakinankaratra	1,946,000	Antsirabe
Vatovavy-Fitovinany	1,529,000	Manakara

* three-four month follow up unable to be completed due to due to adverse weather conditions and pilot sickness.

The two pilot hospitals in Madagascar were used to adapt the prior pilot training course [21] to a three-day format and the Malagasy hospital context, verify translation of materials into Malagasy, and train two local doctors as part of the training team.

From March–August 2015, Mercy Ships and the Ministry of Health jointly contacted each hospital informing its leadership of the rationale for the training course and the dates when checklist training would occur; a letter of support was included, signed by both the Minister of Health and the President of the Ordre des Médecins de Madagascar (the national professional medical society). A needs assessment was also conducted for each hospital via email or written/scanned responses to a survey regarding size of hospital (number of beds), surgical volume, surgical workforce, equipment, current processes (such as checklist use, pulse oximetry, counting of surgical instruments), and any previous training in the checklist. The needs assessment tool was based on a prior pilot study [21].

### Delivery of the training course

During a seven-month period from September 2015 to March 2016, a training team consisting of three to five people visited each of the 21 selected hospitals and taught checklist implementation. The training team always included one or two Malagasy physicians, and one anesthesiologist from a HIC. A total of six different people from HICs plus two Malagasy physicians were members of the training team over the study period. The training team composition was based on a prior pilot study [21] and the numerical composition of 3–5 people was a pragmatic choice based on five people travelling in one vehicle and the need to use a small three-seater plane to access remote hospitals.

Hospital Directors were re-contacted in the weeks before the training team arrived to remind them to ensure the entire surgical team would be available during the training period, and to avoid scheduling elective surgeries during the course. The course was run in each hospital over three days (**Table 3**). The timing and order of the various components remained flexible to accommodate inevitable interruptions for emergency surgical cases, and to accommodate different hospitals start and finish times.

**Table 3:** Outline of the three-day checklist training program.

**Day 1**	• Hospital Director and executive leadership, and Regional Ministry of Health officials invited to the opening of the training course • Introduction to patient safety and the WHO Surgical Safety Checklist including use of film and testimonials
	• Multi-disciplinary workshop to adapt the checklist for the specific local hospital environment. Participants formed small groups to adapt each of the three parts of the checklist, feedback to the whole group and reach consensus.
	• Simulation of adapted checklist in the classroom
**Day 2**	• Specific small group breakout teaching on key components of the checklist ○ Pulse oximeter training, equipment maintenance and hypoxia management ○ Counting of needles, instruments and sponges
	• Further discussion and adaptation of the checklist as necessary
	• Simulations as a whole team, including pulse oximetry and counting, in the operating room.
	• Dinner out with key regional, hospital, and operating room leadership to facilitate further discussion, problem-solving and enlist high-level support.
**Day 3**	• Final discussions and practice using the checklist in theatre with real patients where possible
	• Formal closing ceremony, with Hospital Director and/or Regional Minister of Health for handing over of donated equipment and certificates

All anaesthesia providers were familiar with pulse oximetry from their training but many had not used it since. The pulse oximeter training was detailed and included a post-training assessment to verify knowledge and competency prior to donation of the oximeters. Lifebox pulse oximeters and training materials and assessments were used [27, 28].

Six weeks after the course, one to three senior operating room staff were contacted by telephone to trouble-shoot problems and encourage continued use of the checklist.

### Evaluation at three to four months: Data collection

At three to four months post-training (January to May 2016), an evaluation team visited each hospital for one day. At least one member of the evaluation team had also been a member of the training team. The evaluation team used a mixed methods approach, consisting of anonymous questionnaires, focus group discussion and direct observations to assess implementation of the checklist.

Questionnaires were written in French or Malagasy and used a five-point Likert scale to assess the extent of implementation of the checklist and use of the six basic process measures (confirmation of patient identity and the surgical procedure, assessment of difficult intubation risk, assessment of the risk of major blood loss, antibiotic prophylaxis given within 60 minutes of skin incision, the use of a pulse oximeter, and counting sponges and instruments) [21].

All participants were adults over 18 years of age who gave voluntary, verbal consent to participate. Participants were not paid or otherwise induced to consent.

The primary hypothesis was that by using a three-day team-training program, we could successfully achieve countrywide implementation of the checklist, as measured by at least 50% compliance with all six basic safety processes at three to four months. Therefore the primary outcome measure was number of hospitals using the checklist ‘all the time, in full’.

To test the primary hypothesis, simple descriptive statistics were used. To determine predictors for checklist utilization we used multivariate linear regression to determine the association between covariates and each outcome measure. The primary exposure of interest was use of the surgical checklist. Covariates included number of years participants had practiced medicine, total number of hospital beds, number of surgeries per month. We also aimed to determine associations of checklist use by using the following covariates: verification of patient identify, assessment of difficult intubation risk, assessment of risk of blood loss, prophylactic antibiotics administered within one hour of skin incision, counting sponges, counting needles, counting instruments, and use of a pulse oximeter. For the purpose of multivariate linear regression analysis, Likert responses were scored on a scale of zero to four, and questionnaires with more than one blank answer were excluded from analysis. Those with one blank answer received a score of two for the blank, which is the average score of the zero to four scale used. All analyses were performed using Microsoft Excel. P-values of <0.05 were considered significant.

## Results

427 participants from 21 hospitals in 19 regions received checklist training; median number of participants per site was 18 (range 11–53). Evaluation at three to four months was conducted with 183 participants (most, but not all, of whom had attended the original training) from 20 hospitals in 18 regions. One hospital site was not visited due to adverse weather conditions and pilot sickness. The 183 participants for the evaluation consisted of 33 (18%) surgeons (including obstetricians and ophthalmologists), 34 (18%) anesthetists, 56 (31%) nurses, 12 (7%) surgical assistants, 13 (7%) other (such as stretcher bearers, biomedical technicians and cleaners who assist nurses in the operating room), and 35 (19%) had no title recorded. Median number of years all participants had practiced medicine was four years (range 0.4–33; IQR 2–10). Median number of hospital beds was 77 (range 42–390; IQR 59–129). Median number of surgeries within each hospital was 60 per month (range 27–138; IQR 40.5–80).

Prior to the intervention, no hospital had received checklist training but 2 of 21 hospitals said they had heard of it and tried to use it occasionally. No hospital routinely used pulse oximetry because they all had insufficient pulse oximeters. No hospital routinely counted needles, instruments and sponges or had a count sheet to record this information. No hospital had formal process for discussing the risk or difficult intubation or estimated blood loss or verification of administration of antibiotics. All hospitals reported confirmation of patient identity prior to surgery.

At three to four month follow-up, 119 of 183 (65%) respondents indicated they always used the checklist in full (**Table 4).** With respect to performing the individual six basic safety processes, counting needles and sponges were the processes done most commonly all the time (72% and 70% respectively) and evaluating the risk of difficult intubation was the least commonly reported to be done all the time (54%). Two hospitals had decided to print the checklist and keep the records in the operating room log and produced these completed checklists for each case. Another hospital had painted the counting sheet format on the tiled operating room wall. In several hospitals, the laminated checklists were seen on the table in the operating room or fixed to the wall.

**Table 4:** Frequency of self-reported use of the checklist and the six basic safety processes. Values given as numbers (percentage).

	Always, in full	Always, in part	Sometimes	Occasionally	Never	No response	
**Are you using tde checklist in tde OR**	119 (65%)	24 (13%)	7 (4%)	7 (4%)	1 (1%)	25 (14%)	n = 183
	Always	Most of tde time	Sometimes	Occasionally	Never	No response	
**1. Is the identity of the patient verified with the surgical team before starting surgery**	119 (68%)	25 (14%)	7 (4%)	3 (2%)	0	22 (13%)	n = 176*
**2. Is the risk of difficult intubation evaluated before surgery?**	95 (54%)	27 (15%)	6 (3%)	4 (2%)	4 (2%)	40 (23%)	
**3. Is the risk of large blood loss evaluated before surgery?**	108 (61%)	24 (14%)	6 (3%)	1 (1%)	2 (1%)	35 (20%)	
**4. Is a pulse oximeter used in the OR?**	115 (63%)	20 (11%)	5 (3%)	1 (1%)	0	42 (23%)	n = 183
**5. Are prophylactic antibiotics given before surgery?**	113 (64%)	18 (10%)	9 (5%)	2 (1%)	2 (1%)	32 (18%)	n = 176*
**6a. Are needles/sutures counted before and after surgery?**	131 (72%)	13 (7%)	8 (4%)	0	0	31 (17%)	n = 183
**6b.Are instruments counted before and after surgery?**	114 (62%)	23 (13%)	5 (3%)	0	5 (3%)	36 (20%)	
**6c. Are sponges/swabs counted before and after surgery?**	128 (70%)	16 (9%)	7 (4%)	5 (3%)	2 (1%)	25 (14%)	

* due to an error in printing, four questions were omitted from the evaluation in one hospital (7 participants)

Of the 183 evaluation surveys, 80 were eliminated from multivariate linear regression analysis because they had more than one blank answer. The number of years participants had practiced medicine, total number of hospital beds, and number of surgeries per month were not predictive of checklist use. Checklist use is associated with learning counting instruments (p = <0.05), but is not correlated with the other basic safety processes. (**Table 5**).

**Table 5:** Multivariate linear regression results with checklist use as the dependant variable.

Regression Statistics					
Adjusted R Square	0.200				
Observations	103				
	Coefficients	t Stat	P-value	Lower 95%	Upper 95%
Intercept	1.072	1.422	0.158	-0.426	2.570
Number of years participant in practice	0.007	1.060	0.292	-0.006	0.020
Total number of hospital beds	0.000	0.024	0.981	-0.002	0.003
Number of surgeries per month	0.002	0.513	0.609	-0.005	0.009
Verification of Identity of patient	0.145	1.343	0.183	-0.069	0.359
Assessment of difficult intubation risk	0.156	1.700	0.093	-0.026	0.339
Assessment of risk of blood loss	0.024	0.194	0.847	-0.220	0.268
Prophylactic antibiotics given within 1 hour of skin incision	0.066	0.617	0.539	-0.147	0.280
Counting sponges	0.033	0.198	0.844	-0.299	0.365
Counting needles	-0.108	-0.548	0.585	-0.498	0.283
Counting instruments	0.360	2.897	0.005	0.113	0.607
Use of pulse oximeter	0.003	0.034	0.973	-0.193	0.200

## Discussion

In this paper, we present an evaluation of a three-day nationwide training program to implement the WHO surgical safety checklist in Madagascar. At three to four months post-training, there was wide-spread uptake of the checklist, with 65% of participants using the checklist ‘*always*, *in full’* and 13% *‘always*, *in part’*. Participant’s years of experience practicing medicine, hospital size and surgical volume were not predictive of checklist use.

Since the checklist is known to produce a dose effect, not all aspects of the checklist must be completed to improve patient outcomes [3, 6, 8]. Therefore, it is clinically significant that 78% of participants always use at least part of the checklist. Our study is the first report of wide-scale implementation of the checklist in an LMIC. We did not aim to show a change in patient outcome [5, 7, 19], but rather that widespread implementation was possible in LMICs over a short-time scale. This innovative model, if reproducible in other countries, makes global implementation of the WHO surgical safety checklist a realistic target. Additional benefits of rapid widespread checklist training meant that staff rotating to different regions were still familiar with the checklist on arrival to a new hospital.

Lack of knowledge, lack of resources, and a hierarchical culture are barriers to checklist implementation in LMICs [14, 16, 24]. We aimed to overcome these by dedicating part of the training to learning counting and pulse oximetry; donating pulse oximeters and a laminated copy of the hospitals’ adapted checklist and counting sheets; and encouraging a multidisciplinary approach throughout the implementation. All participants were expected to attend all parts of the training, and demonstrate what they learnt to their colleagues to emphasize teamwork and shared responsibility. Involvement of Chiefs of Service of the operating room, Hospital Directors and the Regional Ministries of Health also aimed to overcome hierarchical barriers. This approach may partially explain our high implementation rate (78%). Since hierarchy is a known barrier, one might expect that participants who have been in practice for longer may be resistant to change and therefore to use of the checklist, or that in larger hospitals with a larger surgical volume, behavior change is harder to achieve. However, our study showed that length of time practicing medicine, hospital size and surgical volume were not predictive of checklist use.

Teamwork is also known to influence surgical outcome [29] and the retention of surgical items [30]. Good counting and documentation is the first step in avoiding retained surgical items, but is rarely done in many LMICs. Similar to other studies [16, 17], our checklist course teaches basic counting and provides a counting sheet on laminated paper. Prior to our study, counting was not routinely performed. Three months after the course, 72%, 62% and 70% of participants now count needles, instruments and sponges, respectively ‘*all the time’*; and 79%, 75%, 79% at least ‘*some of the time’*. Checklist use was associated with counting instruments (p <0.05) but did not correlate with any of the other basic safety process measures. A commonly cited reason for difficult in counting instruments was that the participants couldn’t remember the names of the instruments. Therefore, if participants were motivated enough to learn the names of the instruments, they may have been more motivated to use the checklist, which could account for the correlation between checklist utilization and counting instruments. Other basic safety process (such as pulse oximetry use, administration of prophylactic antibiotics) were behaviors already known to be important by the participants, even if not routinely practiced. Therefore it is not unexpected that these were found to be independent of checklist use, despite the concurrent donation and training in the use of pulse oximeters. All anesthetists were familiar with pulse oximetry from their student training and were aware of the patient safety benefit. However most anesthetists did not used pulse oximetry because oximeters were not commonly available. Therefore one explanation as to why pulse oximetry is not associated with checklist use whereas counting instruments is, is because while checklist training and pulse oximetry training was concurrent, the knowledge and concept of pulse oximetry use was independent of checklist training.

Our study shows the checklist to be always used 65% of the time. Although some basic safety process (risk of difficult intubation, risk of large blood loss, pulse oximeter use, administration of prophylactic antibiotics, instrument counting) are reported as used less frequently than 65% of the time, this is accounted for by the high proportion of ‘no responses’ for those questions. If the ‘no responses’ are excluded then compliance for each of the basic safety process increases to between 70–82%. However, in keeping with the *spirit* of the checklist, which emphasizes teamwork and communication, the number of ‘no responses’ is of concern since all members of the operating team should be aware of each other’s key behaviors. Therefore this remains an area for improvement and future checklist courses should emphasis this aspect of communication.

The follow up rate in our study was 47% (183/427) which is comparable with response rates of 38–49% for surveys and self-reporting studies in high income countries [31, 32]; and higher than other LMIC surgical evaluation studies which report rates of 17–21% [33, 34]. However, the response rate was lower than expected and it became clear during the evaluation phase that while attendance by the whole surgical team was required for the initial training, the same expectation was not outlined for the evaluation. Instead, many hospitals assumed a few participants would be sufficient to report back for the group as a whole. Additionally, although elective operating was cancelled during training, this was not the case during evaluation.

The success of our study may be partly attributable to the strong relationship between Mercy Ships, the Ministry of Health and local healthcare providers, which fostered both a ‘top down’ and ‘bottom up’ approach to our implementation strategy. The strength of relationship between Mercy Ships and local partners maybe a function of Mercy Ships unique position in the country at the invitation of the head of state. However, we would recommend that others considering a nationwide project of this scale invest time in cultivating good relationships at all levels of healthcare from local providers through to government. Additionally, since checklist implementation encourages a style of communication that may challenge existing cultural hierarchies in many contexts, this may present problems that need to be managed at local and national level and prior good trust and working relationships should offset this.

The composition of the training team was based on a prior pilot study [21] which comprised a surgeon, anesthesiologist, operating room nurse, project manager and translator. However the pilot study lacked any local doctors and this was considered a weakness. Therefore for this nationwide implementation we recruited two local doctors who also acted as translators where necessary. We were unable recruit a surgeon to the training team and instead used a medical student with prior experience in governmental policy in LMICs. Lack of surgeon commitment, poor executive leadership and organizational culture, and hierarchy are known barriers to implementation [24, 25]. known barriers to implementation. Despite our lack of surgeon on the training team, we aimed to overcome these barriers by collaborating closely with the government and local providers during project design and implementation, inviting the executive hospital leadership and ministry of health to the opening and closing of the course, and arranging a dinner out with ley leadership figures to facilitate discussion and relationship building.

Other reported barriers to implementation that we aimed to specifically address were lack of understanding of the benefits and evidence-base for the checklist; lack of relevance to the local context and concerns about the time taken to complete the checklist; and lack of sufficient equipment and training [24, 25]. These were addressed through starting the course with an immediate presentation of the scientific evidence coupled with film and testimonials, then multidisciplinary workshops to adapt the checklist to the local environment followed by simulation; and dedicated time taken to address the training and equipment needs identified such as a lack of pulse oximeters and knowledge process to facilitate counting needles, sponges and instruments. This aspect of our study should be able to be replicated by other NGOs and stakeholders aiming to implement nationwide quality improvement projects.

No hospital expressed difficulties at cancelling elective surgery during the three days of training. Most of the hospitals visited only performed elective surgery in the mornings (median number of surgeries per hospital per month = 60; range 27–138; IQR 40.5–80) and not often every day of the week. Therefore there was generally hospital capacity to make up for missed operations in the days after the course.

Our study has a number of limitations. The main limitation is that most of the data were obtained by self-reported questionnaires, and are therefore open to subjective bias. There may be under-reporting in the hope of attaining further help in the form of donations / training; or over-reporting out of a desire to impress or a fear of penalty from hospital directors for non-compliance. Similarly recall may further bias our results. We only recorded individual participants responses to the use of pulse oximetry and counting prior to the study (as part of the specific training sessions in these areas), whereas the other 4 basic safety processes were recorded at a hospital-wide level. Therefore we can only comment on individual improvements in two process measures and hospital-wide improvements in the other four. As far as Mercy Ships and the Ministry of Health were aware there were no other safe surgery initiatives taking place at the time of the study in the hospitals included. Therefore we make the assumption that the changes reported are due to the training intervention and not another concurrent initiative by an alternative NGO in single government hospitals. Since identifying predictors of checklist use was only a secondary outcome, our study may have been underpowered; and also since 80 of 183 responses were excluded due to more than one blank response, this would tend to bias our results towards the centre. Therefore predictors of checklist implementation should be interpreted with caution. Finally, we aimed to measure only change in process, not patient outcome.

Despite these limitations we believe our study has a number of strengths. It was designed to test the hypothesis that a nationwide scale up of checklist implementation was possible, and that this would lead to sustainable change after three to four months. The course was designed to overcome previously known barriers to implementation. We attempted to use both anonymous questionnaires and direct observations to verify responses and reduce the risk of responder bias. Additionally, at least one member of the evaluation team at all times was known to participants which is shown to reduce responder bias in qualitative studies [35, 36] and our responses rates were comparable with other studies [31–34].

In conclusion, our study shows that three to four months after a three-day training course, the WHO surgical safety checklist is used all the time by 65% of participants, ‘always in full’. Participant’s years of practice, hospital size and surgical volume were not predictive of checklist use. Further research is needed to evaluate long-term sustainability, but early results for a three-day training course show promise for widespread checklist implementation and counting instruments in LMICs.

## Supporting information

S1 Dataset (XLSX)
